# DAR (diversity–area relationship): Extending classic SAR (species–area relationship) for biodiversity and biogeography analyses

**DOI:** 10.1002/ece3.4425

**Published:** 2018-09-25

**Authors:** Zhanshan (Sam) Ma

**Affiliations:** ^1^ Computational Biology and Medical Ecology Lab Kunming Institute of Zoology Chinese Academy of Sciences Kunming China; ^2^ Center for Excellence in Animal Evolution and Genetics Chinese Academy of Sciences Kunming China

**Keywords:** diversity–area relationship, diversity–area relationship (DAR) profile, maximum accrual diversity (MAD) profile, pair‐wise diversity overlap (PDO) profile, self‐similarity, species–area relationship

## Abstract

I extend the classic SAR, which has achieved status of ecological law and plays a critical role in global biodiversity and biogeography analyses, to general DAR (diversity–area relationship). The extension was aimed to remedy a serious application limitation of the traditional SAR that only addressed one aspect of biodiversity scaling—species richness scaling over space, but ignoring species abundance information. The extension was further inspired by a recent consensus that Hill numbers offer the most appropriate measures for alpha‐diversity and multiplicative beta‐diversity. In particular, Hill numbers are essentially a series of Renyi's entropy values weighted differently along the rare‐common‐dominant spectrum of species abundance distribution and are in the units of effective number of species (or species equivalents such as OTUs). I therefore postulate that Hill numbers should follow the same or similar law of the traditional SAR. I test the postulation with the American gut microbiome project (AGP) dataset of 1,473 healthy North American individuals. I further propose three new concepts and develop their statistical estimation formulae based on the new DAR extension, including: (i) DAR profile—*z–q* relationship (DAR scaling parameter *z* at different diversity order *q*), (ii) PDO (pair‐wise diversity overlap) profile—*g–q* relationship (PDO parameter *g* at order *q*, and (iii) MAD (maximal accrual diversity: *D*_max_) profile—*D*_max_‐*q*. While the classic SAR is a special case of our new DAR profile, the PDO and MAD profiles offer novel tools for analyzing biodiversity (including alpha‐diversity and beta‐diversity) and biogeography over space.

## 1 INTRODUCTION

The species–area relationship (SAR), well regarded as one of the few classic laws in ecology and biogeography, has been pursued by generations of ecologists and biogeographers since the 19th century (Connor & McCoy, 1979; Drakare, Lennon, & Hillebrand, 2006; Harte, Smith, & Storch, 2009; He & Hubbell, 2011; Helmus, Mahler, & Losos, 2014; Lomolino, 2000; Preston, 1960; Rosenzweig, 1995; Sizling, Kunin, Sizlingova, Reif, & Storch, 2011; Storch, Keil, & Jetz, 2012; Tjørve, 2009; Tjørve & Tjørve, 2008; Triantis, Guilhaumon, & Whittaker, 2012; Watson, 1835). It is hailed as “ecology's most general, yet protean pattern” by Lomolino (2000) and Whittaker and Triantis (2012). SAR relationship had inspired MacArthur and Wilson's (1967) island biogeography theory, and the latter was essential in shifting the focus of ecological research from population to community and in advancing community ecology in the 1970s and after. Today, it still plays a critical role in setting strategy and policies for biodiversity conservation.

Although the study of SAR originated in macroecology of the plants and animals, thanks to the revolutionary genomic and especially metagenomic sequencing technologies, molecular, and microbial ecologists have already joined in the exploration starting approximately a decade ago (Bell et al., 2005; Green et al., 2004; Horner‐Devin, Lage, Hughes, & Bohannan, 2004; Noguez et al., 2005). The revolutionary metagenomic technology has lead to the launches of several national and international research programs, such as European Union's MetaHIT in 2007, US‐NIH human microbiome project (HMP) in 2008, Earth Microbiome Project (EMP) in 2012, and US National Microbiome Initiative (NMI) in 2016 (e.g., Turnbaugh et al., 2007; HMP Consortium (Human Microbiome Project Consortium), 2012, Gilbert, O'Dor, King, & Vogel, 2011). Indeed, the ecological theory has been both a unifying driving force and test bed for this revolution (e.g*.,* Barberán, Casamayor, & Fierer, 2014; Chiu & Chao, 2015; Costello, Stagaman, Dethlefsen, Bohannan, & Relman, 2012; Fierer, 2008; Haegeman et al., 2013; Lozupone, Stombaugh, Gordon, Jansson, & Knight, 2012; Ma, 2015; Ma, Forney, Geng, & Abdo, 2012). Today, molecular ecologists are capable more than ever to test major ecological theories across not only taxa (plants, animals, and microbes) but also ecosystem types (e.g*.,* forest, lakes, ocean, human, and animal microbiomes), and novel findings and insights are revealed more frequently than ever.

In spite of its wide success in biodiversity conservation and biogeography, the classic SAR was limited to the relationship between species richness (the number of species) and area (space). The species abundance was totally ignored in the SAR. Theoretically, there is nothing wrong with this ignorance because it was discovered and verified within its realm (i.e., the scope of species richness). It was not a serious issue either from an application perspective with the datasets from traditional survey technologies for biodiversity and biogeography because species abundance was relatively difficult to obtain until the DNA (RNA) datasets from metagenomic and metagenetic sequencing of environmental samples become readily accessible. Bioinformatics pipelines can be utilized to readily generate the OTU (operational taxonomic unit) tables in the case of 16s‐rRNA amplicon or other metagenetic sequencing. The OTU table contains not only the information about species richness (the number of species) but also the abundance of each species simultaneously. This makes the extension of the classic SAR to general diversity–area relationship (DAR) necessary to fully harness both species richness and abundance information. Indeed, previously, a few group of researchers, notably Helmus and Ives (2012), Mazel et al. (2014), have successfully extended the SAR to phylogenetic and functional diversities. Their extensions not only verified the applicability of SAR models beyond traditional species richness, but also found important applications in identifying more comprehensive conservation hot spots and predicting the impacts of habitat loss. In this study, I extend the SAR to general DAR systematically by adopting the Hill numbers as diversity measures, for both alpha‐diversity and beta‐diversity scaling over space (habitat area), and further propose novel concepts and their quantifications for more effectively and comprehensively analyzing biodiversity and biogeography of the biomes including both macrobiomes and microbiomes.

The choice of Hill numbers for extending the classic SAR was inspired by a recent consensuses that Hill numbers offer the most appropriate measures for alpha‐diversity and multiplicative beta‐diversity partition (Chao, Chiu, & Hsieh, 2012; Chao, Chiu, & Jost, 2014; Ellison, 2010; Jost, 2007). Besides Hill numbers, there have been many diversity indexes (metrics) in existing literature, and two of the most widely used are Shannon entropy (Shannon & Weaver, 1949) and Simpson's (1949) index [*see* Magurran's (2004) monograph for a comprehensive review]. Southwood and Henserson (2000) once commented “the result has been an ‘explosive speciation’ of diversity indices, which initially brought confusion to the subject; in addition, the ubiquitousness of some relationships and the apparent constancy of certain numerical values have added a measure of mystique.” The Hill numbers, which are based on Renyi (1921)’s general entropy, and of which Shannon's entropy is a special case, overcome a significant issue in measuring biodiversity, the influence of rarer species, which made it hardly possible to compare different traditional diversity indexes, such as comparing Shannon index *vs*. Simpson index, a major source for much of the confusion and mystique as critiqued by Southwood and Henserson (2000). In spite of its theoretical soundness, the work of Hill (1973) had not received the attention it deserves until recent years, when Chao et al. (2012, 2014) and Chiu and Chao (2015), Ellison (2010), Jost (2006), Jost (2007) reintroduced the Hill numbers to ecology with additional important clarifications and extensions. Hence, the Hill numbers presented a major conceptual advance in measuring diversity and should be our first choice for extending SAR to DAR.

The SAR has multiple functional forms, often fitting to datasets equally well, although the power law function is predominantly the most often used. Flather (1996) tested nine models; Tjørve (2003, 2009), Dengler (2009), Williams, Lamont, and Henstridge (2009), and Triantis et al. (2012) tested around 20 models, respectively. As those comparative studies often used different classification of sampling or species accrual schemes, the conclusions are frequently debated in the literatures. Traditionally, the modeling strategy has been to use the most parsimonious power function, and preferably its log‐linearized fitting, which facilitates further tractable analysis (e.g., Rosenzweig, 1995). In consideration of the debates on the functional forms of SAR (e.g., He & Hubbell 2012), I test the traditional power law model (PL) as well as what I believe are two most promising extensions, that is, the PL with exponential cutoff (PLEC) and the PL with inverse exponential cutoff (PLIEC). Still I follow the principle of parsimony given more than 20 SAR models exist in the literature (see the excellent review and synthesis such as Tjørve & Tjørve, 2008; Tjørve, 2009; Triantis et al., 2012; Williams et al., 2009), and the excessive computation workload (especially with beta‐diversity scaling) had I tested all of the 20+ models, which is hardly necessary with our objectives set for this study. The taper‐off parameter (*d*) in both PLEC and PLIEC not only addresses a critique to the traditional power law for overestimating diversity (He & Hubbell, 2011), but also preserves the biological interpretations of the scaling parameter (i.e., slope *z* of SAR) as *d* is primarily a revision to the other less biologically meaningful parameter *c* (Tjørve, 2009). Furthermore, I propose to define MAD (maximal accrual diversity) profile, which can be estimated with the PLEC parameters. I also discuss the possible mechanisms of the DAR scaling such as self‐similarity or scale invariance associated with the power law and define a novel pair‐wise diversity overlap (PDO) metric and the PDO profile, based on the inspirations from existing SAR studies (Harte, Kinzig, & Green, 1999; Harte, Blackburn, & Ostling, 2001; Sizling & Storch, 2004; Drakare et al., 2006; and Tjørve & Tjørve, 2008).

To the best of our knowledge, this should be the first extension of the SAR to general diversity–area scaling beyond species richness level in terms of the Hill numbers. The methodological extensions of SAR to general Hill numbers based DAR should not only enrich the theoretical modeling of the diversity scaling in terms of more comprehensive diversity profiles, but also overcome the limitation of the classic SAR. Furthermore, my novel DAR method is applicable to both alpha‐diversity and beta‐diversity. The three new concepts and their statistical parameters including DAR profile, PDO (pair‐wise diversity overlap) profile, and MAD (maximal accrual diversity) profile, developed below, should greatly enrich the quantitative tools for analyzing the biodiversity and biogeography of various biomes on the earth.

## 2 THE METHODS—EXTENDING CLASSIC SAR TO DAR

I use the following definitions and procedures to extend the classic SAR (species–area relationship) to DAR (diversity–area relationship). To save page space, their detailed descriptions are presented in the online Supporting information Appendix S1. The demonstration and interpretation of the DAR definitions and procedures with the AGP datasets are presented in the next section of “demonstrations of the extensions.”

### 2.1 Definitions of alpha and beta diversities

I adopt the Hill numbers to measure both alpha and beta diversities, and multiplicative partition of the Hill numbers to define beta‐diversity.

The Hill numbers, originally introduced as an evenness index from economics by Hill (1973), were reintroduced into ecology by Jost (2007) and Chao et al. (2012) who further clarified Hill's numbers for measuring alpha‐diversity as:

(1) $${}^{q}D={\left(\sum_{i=1}^{S}p_{i}^{q}\right)}^{1/(1-q)}$$

where *S* is the number of species, *p*_*i*_ is the relative abundance of species *i*,* q* is the order number of diversity.

The Hill number is undefined for *q *= 1, but its limit as *q* approaches to 1 exists in the following form:

(2) $${}^{1}D=\lim_{q\to 1}{}^{q}D=\exp\left(-\sum_{i=1}^{S}p_{i}\log\left(p_{1}\right)\right)$$

The parameter *q* determines the sensitivity of the Hill number to the relative frequencies of species abundances. When *q *= 0, the species abundances do not count at all and ^*0*^ *D* = *S*, that is, species richness. When *q *=* *1, ^1^ *D* equals the exponential of Shannon entropy and is interpreted as the number of typical or common species in the community. When *q* = 2, ^2^ *D* equals the reciprocal of Simpson index, that is,

(3) $${}^{2}D=\left(1/\sum_{i=1}^{S}p_{i}^{2}\right)$$

which is interpreted as the number of dominant or very abundant species in the community (Chao et al., 2012). The general interpretation of ^*q*^ *D* (diversity of order *q*) is that the community has a diversity of order *q*, which is equivalent to the diversity of a community with ^*q*^ *D *= *x* equally abundant species.

Recent studies (e.g., Chao et al., 2012; Ellison, 2010; Gotelli & Chao, 2013; Jost, 2007) have advocated the use of multiplicatively defined beta‐diversity, rather than additively defined, by partitioning gamma‐diversity into the product of alpha and beta, in which both alpha (^*q*^ *D*_α_) and gamma (^*q*^ *D*_γ_) diversities are measured with the Hill numbers.

(4) $${}^{q}D_{\beta }={}^{q}D_{\gamma }/{}^{q}D_{\alpha }$$

This beta‐diversity (^*q*^ *D*_β_) derived from the above partition takes the value of 1 if all communities are identical, the value of *N* (the number of communities) when all the communities are completely different from each other (there are no shared species). With Jost (2007) words, this beta‐diversity measures “the effective number of completely distinct communities.” In this article, I compute diversities until *q *= 3, that is, to the third order. Note that a series of the Hill numbers at different order *q* is termed diversity profile (Chao et al., 2012; Jost, 2007).

### 2.2 The DAR models and DAR profiles

As all Hill numbers are in units of species, and in fact, they are referred to as the effective number of species or as species equivalents; intuitively, Hill numbers should follow the same or similar pattern of SAR. I postulate that, similar to the well‐known SAR for species richness (i.e., the Hill number of order zero, ^0^ *D* = *R*), there exist counterparts for the Hill numbers of general *q*‐order, ^*q*^ *D*. I set to investigate the extensions of SAR to general diversity scaling with area (DAR) and further verify our extensions with the AGP dataset.

The basic power function, known as the power law (PL) species scaling law widely adopted in SAR study, is extended to describe the general diversity–area relationship (DAR):

(5) $${}^{q}D=cA^{z}$$

where ^*q*^ *D* is diversity measured in the *q*‐*th* order Hill numbers, *A* is area, and *c* and *z* are parameters.

I also extend two modified PL models for DAR analysis: Power law with exponential cutoff (PLEC) and power law with inverse exponential cutoff (PLIEC) originally introduced to SAR modeling by Plotkin et al. (2000) and Ulrich and Buszko (2003), respectively (also see Tjørve, 2009). The PLEC model is as follows:

(6) $${}^{q}D=cA^{z}\exp\left(dA\right),$$

where *d* is a third parameter and should be negative in DAR scaling models, and exp(*dA*) is the exponential decay term that eventually overwhelms the power law behavior at very large value of *A*. The justification for adding the exponential decay term is because both the human body and the microbial species inhibited on or in human body are finite, and there should be a taper‐off item to reflect the finite size of diversity.

PLIEC is similar to PLEC but with Sigmoid shape, rather than convex as PLEC; it is,

(7) $${}^{q}D=cA^{z}\exp\left(d/A\right)$$

Essentially, PLEC and PLIEC can be considered as extensions to parameter *c*, rather than *z*, that is, *c*(*x*) = *c* exp (*dx*) or *c*(*x*) = *c* exp (*d*/*x*), respectively. Therefore, *z* is assumed to have the similar interpretation as its counterpart in the basic PL. PLEC and PLIEC, however, both behave very differently. The PLEC model asymptotically approaches *cx* ^*z*^ as *x* becomes small, whereas the PLIEC asymptotically approaches *cx* ^*z*^ as *x* becomes large. They were designed to remedy the potentially unlimited accrual of species when the area approaches to infinity by introducing a taper‐off exponent that may even produce asymptote.

I use the following log‐linear‐transformed equations (8), (9), (10) to estimate the model parameters of Equations (5), (6), (7), respectively:

(8) ln(D)=ln(c)+zln(A)

(9) ln(D)=ln(c)+zln(A)+dA

(10) ln(D)=ln(c)+zln(A)+d/A

I consider the ability to fit all three models (PL, PLEC, and PLIEC) in a unified manner—linear transformation—an advantage. I use both linear correlation coefficient (*R*) and *p*‐value to judge the goodness of the model fitting. In fact, either of them should be sufficient to judge the suitability of the models to data. An even more important advantage is that the three models preserve the ecological interpretation of the scaling parameter *z*.

Adopting the convention in SAR analysis, the fitted parameter *z* with Equation (8) is termed the slope of the power law DAR, because *z* represents the slope of the linearized function in log–log space. However, the slope of the DAR as the tangent to the curve in the untransformed axes [i.e*.,* the original PL‐DAR, Equation (5)] is determined by both fitted parameters *z* and *c* as explained in the online Supporting information Appendix S1. This is a significant advantage of the log‐transformed fitting of SAR, and also the primary reason why I adopted the log–log‐linearized fitting in this study.

I define the relationship between DAR model parameter (*z*) of the traditional PL model and the diversity order (*q*), or *z–q* trend, as the DAR profile. It describes the change of diversity scaling parameter (*z*) with the diversity order (*q*), comprehensively. Our definition is obviously inspired by the diversity profile of the Hill numbers (Chao et al., 2012, 2014).

### 2.3 Sampling schemes to fit DAR models

Proper sampling schemes and the accrual of areas are not obvious in our study. I found that Scheiner (2003), Scheiner et al. (2011) type‐III‐B sampling scheme (i.e*.,* no spatial relationship among the areas sampled) is the most appropriate for DAR modeling. Arguments for designing the sampling schemes are provided in the online Supporting information Appendix S1.

Unlike most studies in macroecology, where there is often a natural spatial sequence (or arrangement) among the communities sampled, there is not a naturally occurring spatial sequence (arrangement) among the communities of individual subjects from whom AGP samples were obtained. To avoid the potential bias from an arbitrary order of the community samples, I totally permutated the orders of all the community samples under investigation and then randomly choose 100 (1,000 for alpha‐DAR) orders of the communities generated from the permutation operation. That is, rather than taking a single arbitrary order for accruing community samples in one‐time fitting to the DAR model, I repeatedly perform the DAR model‐fitting 100 (1,000) times with the 100 (1,000) randomly chosen orders. Finally, the averages of the model parameters from the 100 (1,000) times of DAR fittings are adopted as the model parameters of the DAR for the set of community samples under investigation.

### 2.4 The accrual of diversities to fit DAR models

To devise what I believe to be the most appropriate and also natural scheme to accrue diversity, I follow the following three principles. The first is to use the Hill numbers, or what Jost (2007) termed the true diversity; the second is to follow the essence of SAR, as captured by the word “accumulation” or “aggregate,” that is, species (diversity) are accumulated for the accrued areas; the third is that the diversity scaling model should be useful for predicting diversity at different levels of areas accumulated. I consider these three principles as axioms in traditional SAR, and I believe that any extension from SAR to DAR should not violate them. One important advantage for us to stick to the three principles, which are embodied in the traditional SAR theory, is that our new DAR may inherit many of the insights and applications traditional SAR has reveled and offered. The accrual scheme based on the three axioms is described in detail in the online Supporting information Appendix S1.

### 2.5 Predicting MAD (Maximal Accrual Diversity) with PLEC‐DAR models

The wide application of the traditional SAR in the theory and practice of the global biodiversity conservation sets an excellent precedent for the biomedical applications of the DAR models I build in this study. For example, one may use the DAR models predict the (accumulated) diversities in a human population. In the following, I present one novel application—estimation of the maximal accrual diversity (MAD) of the human microbiome with PLEC model. Among the three DAR models, only PLEC may have a maximum, as derived below based on PLEC model of DAR.

The necessary condition for Equation (6) to achieve maximum is its derivative equals zero, that is,

$$\frac{df(A)}{\mathrm{dA}}={\left({}^{q}D\right)}^{\prime }={\left[cA^{z}\exp\left(dA\right)\right]}^{\prime }=0$$

$$\begin{array}{ccc} & & czA^{z-1}\exp\left(dA\right)+cA^{z}\exp\left(dA\right)d=0\\ & & czA^{z-1}+cA^{z}d=0\;\left(c\neq 0\right)\\ & & zA^{z-1}+A^{z}d=0\\ & & z+Ad=0 \end{array}$$

Hence, when

(11) $$A_{\max}=-z/d$$

^*q*^ *D* may have a maximum in the following form:

(12) $$\text{Max}\left({}^{q}D\right)=c{\left(-\frac{z}{d}\right)}^{z}\exp\left(-z\right)=cA_{\max}^{z}\exp\left(-z\right)$$

Eqs. (11) and (12) can be utilized to predict the maximal accrual diversity (MAD) of the human microbiome, whether it is alpha‐ or beta‐diversity. I define the MAD profile as the relationship between the *D*_max_ and diversity order *q*, that is, *D*_max_–*q* trend. It is noted that in the above derivation, there are two implicit assumptions: One is that *A*_max_ > 0, which requires *z* and *d* of different signs, and another is *z *>* *0, *d* < 0. The situation restricted by the first assumption is ecologically meaningless, and I can safely eliminate it from consideration because negative accrual (*A*_max_ < 0) is not possible. The situation restricted by the second assumption (i.e*., z *< 0 & *d *> 0) is possible both mathematically and ecologically, but the extreme value is then minimum rather than maximum. In the case of the traditional SAR, the *z *< 0 is not justified. However, in general DAR with Hill numbers, *z *< 0 is possible at higher diversity orders. In this study, I use the average *z* and *d* from 100/1,000 times of resampling operations, to compute *D*_max_. In case the average *z* and *d* do not satisfy the above two assumptions, I select the valid permutations from 100/1,000 re‐samplings, compute *D*_max_ for each valid permutation, and then obtain the average *D*_max_ of the valid permutations.

### 2.6 The self‐similarity property and pair‐wise diversity overlap (PDO) profile

As diversity measured in Hill numbers are the numbers of species equivalents, I expect that the PL‐DAR should possess the self‐similarity or scale invariance as SAR has demonstrated (Drakare et al., 2006; Harte et al., 1999, 2001; Sizling & Storch, 2004 and Tjørve & Tjørve, 2008). Adopting similar derivation process with the SAR, the following properties of PL‐DAR can be worked out as follows:

From Equation (5), the following equations can be derived as follows:

(13) dD/dA=zD/A

(14) $$\frac{dD/D}{dA/A}=z$$

Hence, *z* is the ratio of diversity accrual rate to area increase rate.

By setting *A* = 1, *S*_*0*_ = *cA* ^*z*^ = *c;* hence, *c* is the number of species equivalents of diversity in one unit of area, but not per unit of area because the scaling is nonlinear.

The self‐similarity is also known as scale invariant, which refers to the following mathematical property of the power law:

(15) $$f\left(\alpha A\right)=c{\left(\alpha A\right)}^{z}=\alpha^{z}f\left(A\right)\propto f\left(A\right)$$

that is, scaling the argument *A* (area) by a constant factor α is equivalent to scaling its function proportionally by a constant factor α^*z*^ Therefore, all power laws with a particular scaling exponent *z* are equivalent up to constant factors because each is a scaled version of the others. The scale invariance is also responsible for the linear relationship after log‐transformation of power law (Equation (8)), and the resulted straight line on log–log plot is termed the signature of power law. This is another reason I adopted log–log‐linear transformation fitting of the power law; of course, this is essentially the same argument I argued previously (i.e., the “slope” argument).

From (15), it is also obvious that:

(16) $$D_{\alpha A}/D_{A}=\alpha^{z}$$

where *D*_α*A*_ and *D*_*A*_ are the diversity at area size α*A* and *A*, respectively, α^*z*^ is the scaling factor. I omitted diversity order (*q*) to simplify the notation, for example, *D*_*A*_ in place of ^*q*^ *D*_*A*_.

Applying log function with the base (α) on both sides of (16), there is

(17) $$\log_{\alpha }\left(D_{\alpha A}/D_{A}\right)=\log_{\alpha }\alpha^{z}=z\log_{\alpha }\alpha =z$$

It follows that

(18) $$D=cA^{\log_{\alpha }\left(D_{\alpha A}/D_{A}\right)}$$

If *α *= 2, then *z* = log _2_(*D*_2*A*_/*D*_*A*_)

(19) $$D=cA^{\log_{2}}\left(D_{2A}/D_{A}\right)$$

is a special case of (18).

The fraction (*h*) of new diversity due to expansion of *α* times of original area *A* can be expressed as:

(20) $$h=\left(D_{\alpha A}-D_{A}\right)/D_{A}=\alpha^{z}-1$$

Similarly, the proportion of new diversity in the *j*‐th area (of the same size) added can be computed with the following equation:

(21) $$h_{j}=\left(D_{\mathrm{jA}}-D_{(j-1)A}\right)/D_{A}=j^{z}-{\left(j-1\right)}^{z}$$

Tjørve and Tjørve (2008) termed α as area multiplication rate*,* and I adopt the same term for DAR, and *h* is the fraction of new diversity accumulated as a function of *z*. When α = 2, the proportion of new diversity *h* = 2^*z* − 1^, the diversity overlap (*g*) of two bordering areas of the same size (computed as the proportion of the new diversity in the second area) is as:

(22) $$g=\left(2D_{A}-D_{2A}\right)/D_{A}=2-2^{z}$$

In (22), *g* is also the scale‐invariant overlap because it is the overlap between two areas of the *same* size.

If *z *=* *1, then *g *=* *0, no overlap; and if *z *=* *0, *g *=* *1, totally overlap. In reality, *g* should between 0 and 1.

As the equal size of area assumption is largely true in the case of sampling human microbiome, the parameter *z* of the PL‐DAR can be utilized to estimate the pair‐wise diversity overlap (PDO), that is, diversity overlap between two individuals, in the human microbiome with Equation (22). Given the range of *g* is between 0 and 1, I may even use percentage notation to measure pair‐wise diversity overlap.

Similar to previous definitions for DAR profile (*z–q* pattern) and MAD profile (*D*_max_–*q* pattern), I define PDO profile (*g–q* pattern) as a series of values of the pair‐wise diversity overlap metric (*g*) at different diversity order (*q*). The profile comprehensively (at different diversity order or nonlinear level, *q*) captures the average‐level, pair‐wise overlap (similarity) between two communities in a meta‐community setting. Although the *g* (PDO profile) is simply a precise function of PL‐DAR *z* (DAR profile) (equation (22)), the former is far more convenient for measuring community overlap (similarity), which should have more straightforward and intuitive usage.

## 3 DEMONSTRATIONS OF THE EXTENSIONS

### 3.1 The American Gut microbiome project (AGP) dataset

I use the datasets from the American Gut Project (AGP: http://americangut.org/), part of the Earth Micorbiome Project (EMP). The dataset of OTU tables (which are equivalent to the species abundance data of a community in macroecology and utilized to test the DAR extensions throughout this article), were rarefied to 10,000 sequence reads per sample computed from the DNA‐sequencing data of the 16s‐rRNA (v4 region) marker genes from the gut microbiome of 6,500 volunteer participants (as of October 2015), was downloaded from the AGP website (https://github.com/biocore/American-Gut/tree/master/data/AG). According to AGP website (http://americangut.org/about/), the protocols used by the AGP project to process the samples and obtain the OTU tables have been extensively tested and benchmarked by Knight Lab at the University of California, San Diego, one of the largest microbiome research laboratories in the world. I selected the dataset of 1,473 healthy Caucasian individuals and excluded the samples from individuals with IBD, diabetes, and any other diseases.

**Figure 1:**
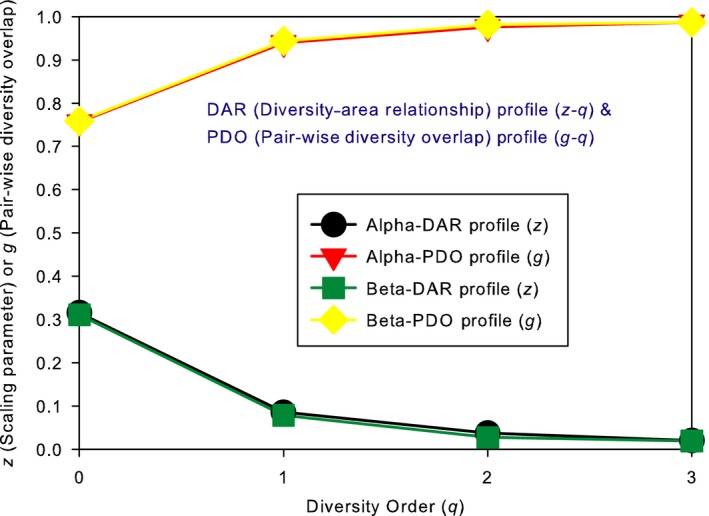
The DAR profile and PDO profile for the alpha‐diversity and beta‐diversity built with the AGP dataset: (*i*) The alpha‐DAR profile (*z–q*) and beta‐DAR profile (*z–q*) are nearly overlapped, and similarly the alpha‐PDO profile (*g–q*) and beta‐PDO profile (*g–q*) are nearly overlapped; (*ii*) The DAR profile is monotonically decreasing with diversity order (*q*), and the PDO profile is monotonically increasing with *q*

The test of DAR extensions with the AGP dataset consists of two parts: alpha‐DAR and beta‐DAR modeling, each with three DAR models, PL, PLEC, and PLIEC, respectively. I further define DAR, MAD, and PDO profiles for the alpha‐ and beta‐diversity scaling of the human gut microbiome, respectively. Tables 1 and 2 list the alpha‐DAR models, and Table 3 lists the beta‐DAR models. Figure 1 illustrates the DAR and PDO profiles for alpha and beta diversities, and Figure 3 illustrates the alpha‐MAD profile and beta‐MAD profile, respectively.

**Table 1:** Fitting the alpha‐DAR (diversity–area relationship) models with 100 times of resampling of 1,473‐Subjects AGP datasets

Diversity order and statistics		Power law (PL)						PL with inverse exponential cutoff (PLIEC)						PL with exponential cutoff (PLEC)							
		*z*	ln(*c*)	*R*	*p*‐Value	*g*	*N* a	*z*	*d*	ln(*c*)	*R*	*p*‐Value	*N* a	*z*	*d*	ln(*c*)	*R*	*p*‐Value	*N* a	*A*_max_	*D*_max_
*q = 0*	Mean	0.315	6.908	0.986	0.000	0.756	100	0.291	−1.493	7.067	0.996	0.000	100	0.387	−0.0002	6.593	0.995	0.000	100	1,994	9407.4
	Std. Err.	0.001	0.005	0.000	0.000	0.001		0.001	0.024	0.006	0.000	0.000		0.001	0.0000	0.007	0.000	0.000			
	Min	0.297	6.775	0.975	0.000	0.742		0.271	−2.211	6.950	0.990	0.000		0.347	−0.0003	6.387	0.988	0.000			
	Max	0.332	7.023	0.993	0.000	0.771		0.308	−0.946	7.214	0.999	0.000		0.429	−0.0001	6.809	0.999	0.000			
*q = 1*	Mean	0.085	4.849	0.789	0.000	0.939	100	0.058	−1.677	5.027	0.930	0.000	100	0.165	−0.0002	4.504	0.900	0.000	100	775	229.2
	Std. Err.	0.002	0.014	0.009	0.000	0.001		0.002	0.054	0.016	0.004	0.000		0.003	0.0000	0.018	0.006	0.000			
	Min	0.044	4.475	0.529	0.000	0.900		0.010	−3.350	4.624	0.816	0.000		0.081	−0.0004	4.100	0.654	0.000			
	Max	0.138	5.130	0.943	0.000	0.969		0.115	−0.599	5.357	0.987	0.000		0.239	−0.0001	4.935	0.988	0.000			
*q = 2*	Mean	0.037	3.585	0.508	0.000	0.976	90	0.014	−1.207	3.740	0.763	0.000	100	0.086	−0.0001	3.386	0.664	0.000	99	622	47.0
	Std. Err.	0.003	0.020	0.027	0.000	0.002		0.003	0.061	0.023	0.014	0.000		0.005	0.0000	0.026	0.023	0.000			
	Min	−0.012	3.040	0.061	0.000	0.917		−0.047	−2.764	3.125	0.431	0.000		−0.017	−0.0003	2.825	0.080	0.000			
	Max	0.115	3.959	0.955	0.019	1.008		0.102	0.035	4.191	0.971	0.000		0.193	0.0001	3.919	0.976	0.009			
*q = 3*	Mean	0.020	3.045	0.465	0.000	0.987	94	0.005	−0.846	3.143	0.667	0.000	100	0.052	−0.0001	2.907	0.601	0.000	100	586	24.3
	Std. Err.	0.003	0.022	0.024	0.000	0.002		0.003	0.060	0.023	0.020	0.000		0.005	0.0000	0.027	0.022	0.000			
	Min	−0.030	2.512	0.057	0.000	0.931		−0.056	−2.235	2.512	0.109	0.000		−0.064	−0.0003	2.308	0.083	0.000			
	Max	0.096	3.423	0.956	0.027	1.021		0.096	0.566	3.584	0.961	0.000		0.169	0.0002	3.517	0.963	0.006			

a The failed fitting cases (100−*N*) were removed to compute the statistics of the model parameters, but the major results such as the mean of the model parameters have little differences from the results without removing the failed fitting cases (see Supporting information Table S1 in Appendix S1). Supporting information Table S4 in Appendix S2 listed the 100 alpha‐DAR models from the 100 times of resampling.

**Table 2:** Fitting the alpha‐DAR (diversity–area relationship) models with 1,000 times of resampling of 1,473‐Subjects AGP datasets

Diversity order and statistics		Power law (PL)						PL with inverse exponential cutoff (PLIEC)						PL with exponential cutoff (PLEC)							
		*z*	ln(*c*)	*R*	*p*‐Value	*g*	*N* a	*z*	*d*	ln(*c*)	*R*	*p*‐Value	*N* a	*z*	*d*	ln(*c*)	*R*	*p*‐Value	*N* a	*A*_max_	*D*_max_
*q = 0*	Mean	0.314	6.911	0.986	0.000	0.757	1,000	0.290	−1.508	7.071	0.996	0.000	1,000	0.386	−0.0002	6.598	0.995	0.000	1,000	2,006	9413.6
	Std. Err.	0.000	0.002	0.000	0.000	0.000		0.000	0.008	0.002	0.000	0.000		0.000	0.0000	0.002	0.000	0.000			
	Min	0.291	6.771	0.954	0.000	0.739		0.262	−3.261	6.917	0.989	0.000		0.336	−0.0003	6.295	0.978	0.000			
	Max	0.335	7.081	0.995	0.000	0.777		0.312	−0.847	7.269	0.999	0.000		0.456	−0.0001	6.866	0.999	0.000			
*q = 1*	Mean	0.082	4.870	0.784	0.000	0.942	1,000	0.054	−1.714	5.051	0.935	0.000	1,000	0.157	−0.0002	4.542	0.890	0.000	1,000	780	228.6
	Std. Err.	0.001	0.004	0.003	0.000	0.000		0.001	0.020	0.005	0.001	0.000		0.001	0.0000	0.007	0.002	0.000			
	Min	0.016	4.438	0.350	0.000	0.895		−0.007	−5.275	4.568	0.738	0.000		0.022	−0.0004	3.861	0.412	0.000			
	Max	0.144	5.286	0.958	0.000	0.989		0.125	−0.339	5.469	0.993	0.000		0.290	0.0000	5.263	0.993	0.000			
*q = 2*	Mean	0.030	3.635	0.469	0.001	0.980	948	0.009	−1.224	3.775	0.749	0.000	1,000	0.072	−0.0001	3.454	0.638	0.000	995	616	46.8
	Std. Err.	0.001	0.007	0.008	0.000	0.001		0.001	0.023	0.007	0.005	0.000		0.002	0.0000	0.010	0.007	0.000			
	Min	−0.068	2.942	0.052	0.000	0.905		−0.088	−4.339	3.025	0.083	0.000		−0.102	−0.0004	2.570	0.079	0.000			
	Max	0.131	4.301	0.956	0.047	1.046		0.118	0.499	4.438	0.983	0.006		0.245	0.0002	4.403	0.966	0.010			
*q = 3*	Mean	0.014	3.083	0.429	0.001	0.991	929	−0.001	−0.861	3.181	0.655	0.000	997	0.039	−0.0001	2.977	0.577	0.000	996	561	24.2
	Std. Err.	0.001	0.007	0.008	0.000	0.001		0.001	0.023	0.007	0.007	0.000		0.002	0.0000	0.010	0.007	0.000			
	Min	−0.098	2.345	0.052	0.000	0.914		−0.108	−3.746	2.403	0.071	0.000		−0.153	−0.0004	2.028	0.078	0.000			
	Max	0.119	3.842	0.957	0.048	1.065		0.112	0.901	3.906	0.976	0.025		0.213	0.0003	3.985	0.971	0.012			

a The failed fitting cases (1,000−*N*) were removed to compute the statistics of the model parameters, but the major results such as the mean of the model parameters have little differences from the results without removing the failed fitting cases (see Supporting information Table S2 in Appendix S1). Supporting information Table S5 in Appendix S2 listed the 1,000 alpha‐DAR models from the 1,000 times of resampling.

**Table 3:** Fitting the beta‐DAR (diversity–area relationships) models with 100 times of resampling of 1473‐subjects AGP datasets

Diversity order and statistics		Power law (PL)						PL with inverse exponential cutoff (PLIEC)						PL with exponential cutoff (PLEC)							
		*z*	ln(*c*)	*R*	*p*‐Value	*g*	*N* a	*z*	*d*	ln(*c*)	*R*	*p*‐Value	*N* a	*z*	*d*	ln(*c*)	*R*	*p*‐Value	*N* a	*A*_max_	*D*_max_
*q = 0*	Mean	0.311	0.971	0.990	0.000	0.759	100	0.283	−2.236	1.155	0.997	0.000	100	0.377	−0.0002	0.683	0.996	0.000	100	2,056	24.2
	Std. Err.	0.001	0.006	0.000	0.000	0.001		0.001	0.024	0.008	0.000	0.000		0.001	0.0000	0.008	0.000	0.000			
	Min	0.285	0.781	0.981	0.000	0.732		0.251	−2.848	0.963	0.992	0.000		0.338	−0.0003	0.475	0.993	0.000			
	Max	0.343	1.148	0.994	0.000	0.781		0.314	−1.789	1.376	1.000	0.000		0.417	−0.0001	0.885	0.999	0.000			
*q = 1*	Mean	0.078	1.167	0.808	0.000	0.944	100	0.048	−2.429	1.367	0.951	0.000	100	0.145	−0.0002	0.878	0.910	0.000	100	782	5.5
	Std. Err.	0.002	0.015	0.011	0.000	0.002		0.003	0.048	0.017	0.003	0.000		0.003	0.0000	0.019	0.007	0.000			
	Min	0.022	0.833	0.439	0.000	0.908		−0.011	−3.742	0.963	0.871	0.000		0.055	−0.0004	0.502	0.595	0.000			
	Max	0.126	1.554	0.957	0.000	0.984		0.107	−1.412	1.806	0.987	0.000		0.213	0.0000	1.410	0.993	0.000			
*q = 2*	Mean	0.027	1.112	0.577	0.000	0.981	97	0.004	−1.787	1.265	0.770	0.000	100	0.074	−0.0001	0.908	0.703	0.000	98	610	3.7
	Std. Err.	0.004	0.029	0.024	0.000	0.003		0.005	0.094	0.033	0.015	0.000		0.006	0.0000	0.035	0.021	0.000			
	Min	−0.073	0.545	0.072	0.000	0.919		−0.119	−4.999	0.604	0.122	0.000		−0.099	−0.0004	0.177	0.105	0.000			
	Max	0.112	1.826	0.937	0.010	1.050		0.102	0.438	2.124	0.945	0.000		0.195	0.0002	1.823	0.967	0.000			
*q = 3*	Mean	0.019	1.081	0.555	0.000	0.987	99	−0.001	−1.534	1.209	0.701	0.000	100	0.061	−0.0001	0.883	0.688	0.000	97	605	3.4
	Std. Err.	0.005	0.037	0.024	0.000	0.004		0.006	0.125	0.043	0.017	0.000		0.009	0.0000	0.047	0.021	0.000			
	Min	−0.123	0.404	0.060	0.000	0.914		−0.178	−6.124	0.478	0.205	0.000		−0.192	−0.0005	0.040	0.129	0.000			
	Max	0.119	1.984	0.923	0.020	1.082		0.107	1.008	2.389	0.933	0.000		0.209	0.0004	2.272	0.958	0.000			

a The failed fitting cases (100−*N*) were removed to compute the statistics of the model parameters, but the major results such as the mean of the model parameters have little differences from the results without removing the failed fitting cases (see Supporting information Table S3 in Appendix S1). Supporting information Table S6 in Appendix S2 listed the 100 beta‐DAR models from the 100 times of resampling.

### 3.2 Alpha‐DAR analysis

Tables 1 and 2 listed the test results of the alpha‐DAR modeling with 100 and 1,000 times of resampling, respectively. Table 3 listed the test results of the beta‐DAR modeling with 100 times of sampling. In these tables, I listed the following: the diversity order (*q*) in Hill numbers, model parameters (*z*, ln*c*,* d*), *R* (linear correlation coefficient), *p*‐value measuring the goodness of the model fitting, pair‐wise diversity overlap (*g*), and the number of successful fitting of DAR models (*N*). Listed in the last two columns of the PLEC models are the theoretical maximal accrual diversity (MAD) (*D*_max_) and corresponding area accrual (*A*_max_), predicted with PLEC model (Equations (11), (12)).

From both Tables 1 and 2, I expose the following findings regarding the test of alpha‐DAR models.

#### 3.2.1 The performance of alpha‐DAR models

The number of successful fittings (*N*) shows that at lower diversity order *q *=* *0 & 1, all three DAR models fitted to the AGP dataset successfully (*p* < 0.0001) in 100% of the sampled cases in both 100 and 1,000 times of resampling operations. At high diversity order *q *= 2 & 3, the PLEC and PLIEC succeeded in 99% sampling cases, and both the models performed slightly better than the PL model (90%–95%) (*p *< 0.01). The linear correlation coefficients (*R*) confirmed the finding. For example, with PL model, at lower diversity order, *R* ranges between 0.94 and 0.99, and at higher diversity order, *R* ranges between 0.47 and 0.51. The decreased goodness‐of‐fit is expected as the higher‐order Hill numbers have relatively stronger nonlinearity. Although either *p*‐value or *R* alone is sufficient to show the model fitting, I present both to show more comprehensive information (*R* showing the level of linear correlation). I conclude from the above finding that the extension of SAR to alpha‐DAR (in the Hill numbers) with three DAR models is fully justified and verified with the AGP dataset, a single largest HMP dataset I am aware of. All three models are sufficient to describe alpha‐DAR, and the PL model is preferred if one is in favor of the principle of parsimony. PLIEC performed the best, but PLEC has an advantage over the other two models in predicting the MAD and establishing the MAD profile—*D*_max_–*q* pattern. The finding also shows that 100 times of resampling operations are enough to deal with the random noise from arbitrarily setting the accrual order of individuals, given the results from both 100 and 1,000 times of samplings had little difference.

I now discuss a potential complication arisen from extending SAR to DAR, that is, negative scaling parameter (*z*) at higher diversity order *q *= 2–3. Table 4 below listed the number of negative *z*‐values or positive *d*‐values (to be discussed later) from fitting the three DAR models. The percentages of negative *z* of the three models PL, PLIEC, and PLEC at *q *=* *2 for alpha‐diversity DAR are 11%, 37%, and 5%, respectively, and at *q *=* *3, 30%, 44% and 12%, respectively. As these percentages numbers were computed from 100 (1,000) times of DAR models from resampling of the permutation orders of a single dataset, rather than multiple datasets, I consider the negative *z* was largely due to arbitrary ordering for diversity accrual, which is also the very reason why I adopt the average of 100 times of resampling. If the average *z* from the 100 (1,000) times of reordering (resampling from total permutations of the 1,473 individual in AGP dataset) is positive, I still consider the DAR model for the AGP dataset as positive DAR scaling.

**Table 4:** The percentages of negative *z*‐values or positive *d*‐values in the DAR models with 100 (1,000) times of resampling from the random permutations of 1,473 individuals in the AGP datasets

Diversity order	Model	Alpha‐DAR (100 times)		Alpha‐DAR (1,000 times)		Beta‐DAR (100 times)	
		%Negative *z*	%Positive *d*	%Negative *z*	%Positive *d*	%Negative *z*	%Positive *d*
*q* = 0	PL	0	NA	0	NA	0	NA
	PLIEC	0	0	0	0	0	0
	PLEC	0	0	0	0	0	0
*q *= 1	PL	0	NA	0	NA	0	NA
	PLIEC	0	0	0.7	0	4.0	0
	PLEC	0	0	0	0	0	0
*q *= 2	PL	11.1	NA	13.3	NA	23.7	NA
	PLIEC	37.0	2.0	39.8	2.50	40.0	1.0
	PLEC	5.00	5.0	11.3	11.3	13.7	13.7
*q *= 3	PL	29.8	NA	35.1	NA	29.3	NA
	PLIEC	44.0	8.0	53.8	11.2	42.0	9.0
	PLEC	12.0	12.0	22.5	22.5	21.6	21.6

Of course, I need to answer a more fundamental question, are negative *z*‐values justified ecologically? Our answer is yes. This is because at higher diversity orders, unlike species richness, diversity does not necessarily rise in an accrued assemblage (community). For example, rare species in individual assemblage may become commoner, rarer or the same level of rareness when the assemblage is pooled together with another assemblage. Consequently, the diversity of the pooled community could be up, down, or unchanged. As a side note, as mentioned previously, as parameter *d* in PLEC and PLIEC is an extension to *c*, rather than *z*, parameter *z* should have similar ecological interpretations as in the original PL model. Therefore, I consider negative *z* in the three DAR models as an ecological reality, rather than a mathematical artifact. In the case of AGP dataset, I adopt the average *z* of 100 times resampling of the permutation orders because there is not a natural order to accrue the diversity. If there is a natural order for accruing the diversity, that order should be followed to fit the DAR model, and the sign of *z* should be determined by the natural order.

An additional issue, similar to the sign of *z*, is the sign of *d* in PLEC and PLIEC. In both PLEC and PLIEC, *d* as an exponential cutoff parameter is usually negative. However, when *z *<* *0, it is possible that *d *>* *0. This has an implication for computing MAD (*D*_max_), as in explanation for Equations (11) and (12) in previous section on the derivation of MAD. Indeed, as shown in Table 4, in the case of PLEC, negative *z* is always matched with positive *d*.

Yet another interesting finding can be observed from Tables 1, 2, and 4 (also Supporting information Tables S4‐S6), while PLIEC has the best statistical fitting judged from *p*‐value and *R*, followed by PLEC and PL, PLEC has the lowest numbers of negative *z,* followed by PL and PLIEC. If I consider negative *z* a potential issue, although which may not be an issue at all as explained previously, PLEC seems to have an advantage of the lowest percentages of negative *z*‐values, besides being able to predict MAD. The advantage of PL model is its simplicity and established ecological interpretations, but it may fail to fit DAR data at higher diversity orders. Table 4 also suggests that PLIEC has the highest percentage of negative *z*‐values, and yet, negative *z*‐values are not matched with positive *d*‐values as in the case of PLEC. I am concerned that, although PLIEC has the best statistical fitting, its behavior may be unnecessarily more complicated than the PL and PLEC models. In consideration of the findings discussed above, I recommend the utilization of PL for DAR profile and PDO profile, and PLEC for MAD profile, at least for the study of human microbiome.

#### 3.2.2 The parameter ranges of alpha‐DAR models

In all three alpha‐DAR models, the scaling exponent (*z*) decreases with the increase in the diversity order (*q*). The alpha‐DAR profile, that is, the *z–q* series with the PL model is [0.315, 0.085, 0.037, 0.020]. The counterpart series for PLIEC and PLEC are [0.291, 0.058, 0.014, 0.005], and [0.387, 0.165, 0.086, 0.052], respectively. Hence, the alpha‐DAR profile is a monotonically decreasing curve (Figure 1). As existing literature has not established a systematic range for the diversity scaling parameter (*z*) beyond species richness, comparison with existing studies is limited to *zero*‐order alpha‐diversity (i.e*.,* SAR). According to Green and Bohannan's (2006) review, the reported SAR exponents in microbes were in the range between 0.019 and 0.470, but most values were below 0.2 (eight of 11 studies). Peay, Bruns, Kennedy, Bergemann, and Garbelotto (2007) reported a range of 0.20‐0.23 eukaryotic soil microbes. A major limitation of these early pioneering studies on the testing of SAR with microbes is then low throughput of DNA‐sequencing technology in detecting bacteria, and consequently, the diversity and SAR exponent may be significantly underestimated. Recent studies further confirmed the validity of microbial SAR (e.g., van der Gast, 2013, 2015; Ruff et al., 2015). As to the range of *z*‐value in plants and animals in macroecology literature, there are many reports but most pointed to a range between 0.2 and 0.4. A more recent large‐scale investigation with 601 datasets from terrestrial islands by Triantis et al. (2012) revealed a full range from 0.064 to 1.312 with 51% fell between 0.2% and 0.4%, 25% exceeded 0.4, and an average of *z *=* *0.321. Our study hence not only falls in the general range, but also happens to be rather close to the average (0.315 vs. 0.321) reported in macroecology.

#### 3.2.3 Alpha‐MAD profile prediction

**Figure 2:**
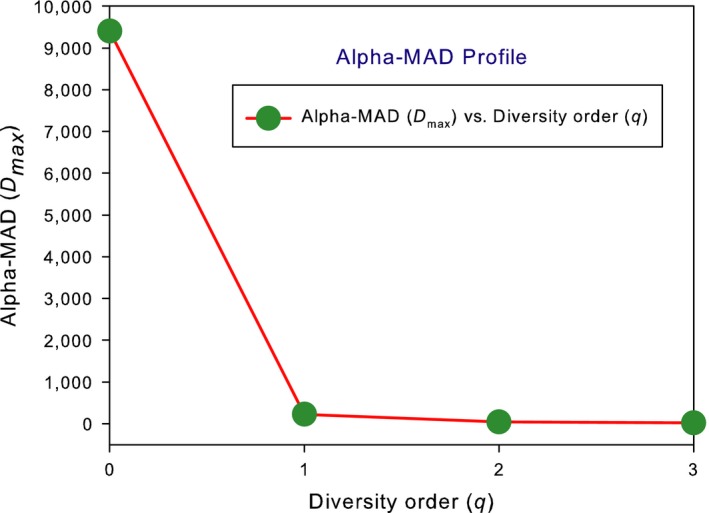
The alpha‐MAD profile for the alpha‐diversity built with the AGP dataset: The MAD profile is monotonically decreasing with diversity order (*q*)

The last two columns in Tables 1 and 2 listed the alpha‐MAD profile or *D*_max_–q predicted by the alpha‐DAR PLEC models, that is, *D*_max_ = [9,434, 229.7, 47.4, 24.5] (Figure 2) and *A*_max_ = [2,028, 802, 969, 1,135] for (*q *=* *0, 1, 2, 3). I consider the prediction of *D*_max_ series rather reasonable based on the existing reports on species richness in the human gut microbiome (HMP Consortium (Human Microbiome Project Consortium) (2012). Nevertheless, I am somewhat reserved with the estimates of *A*_max_, the number of individuals (‘area’) corresponding to the MAD, which seems being influenced by the random noise in the process of area/diversity accrual. This is evidenced by the wide range (max–min) of *A*_max_ in Tables 1 and 2, but the corresponding *D*_max_ estimates are rather robust as indicated by their rather narrow ranges.

#### 3.2.4 Pair‐wise alpha‐diversity overlap

Based on the self‐similarity property of PL‐DAR, I introduce a new metric, pair‐wise diversity overlap (PDO) (*g*) and PDO profile, as derived previously. The *g*‐series (*q *=* *0–3) or PDO profile for the alpha‐DAR is [0.756, 0.939, 0.976, 0.987] (Figure 1). While the interindividual (interpersonal) similarity at the species richness level (*q *=* *0) can be relatively low (0.756% or 75.6%), due to functional redundancy, the similarity at higher diversity levels (*q *>* *0) should be rather high (94%–99%), which explains the observed monotonically increasing pattern of PDO profile.

#### 3.2.5 Summary on the alpha‐DAR

I reiterate the following four important findings regarding the alpha‐DAR scaling: First, extending the SAR to alpha‐DAR measured in the Hill numbers is appropriate as verified with the AGP dataset. PL‐DAR model is preferred in consideration of its simplicity and established ecological interpretations in the literature. PL‐DAR parameter *z* is the diversity accrual rate to area increase rate or the slope of the linear‐transformed PL model. Parameter *c* is the number of species equivalents of diversity in one unit of area (but not per unit of area) as the scaling is nonlinear. Due to the interindividual heterogeneity (variability), *c* may be strongly influenced by the accrual order (what I termed random noise). It is mainly for this reason that I performed 100/1,000 times of resampling operations and computed the averages from sampling to get the DAR model parameters. I also found that 100 times of sampling is enough to get reliable model parameters. Second, the alpha‐DAR profile for *q *=* *0–3 is *z* = [0.315, 0.085, 0.037, 0.020], monotonically decreasing with the diversity order (*q*). The parameter (*z*) at species richness level (*q *=* *0) of AGP not only falls in the range of Triantis et al. (2012) meta‐analysis, but also approaches to the average they reported in macroecology (AMGP = 0.315 *vs*. Triantis meta‐analysis = 0.321). Third, the PLEC = DAR model can be harnessed to predict *the* alpha‐MAD profile for *q *=* *0–3, *D*_max_ = [9,434, 229.7, 47.4, 24.5]. This is essentially the theoretical maximal accrual diversity of the human gut micorbiome, estimated from the AGP dataset. Fourth, based on the self‐similarity property, the pair‐wise diversity overlap (*g*) between two individual samples (two humans in AGP case) or the alpha‐PDO profile for *q *=* *0 to 3 is *g *= [0.756, 0.939, 0.978, 0.987]. This metric is obviously useful for characterizing the *average* pair‐wise similarity (dissimilarity) between two human individuals in their gut microbiome diversity. Although other ecological similarity measures (e.g., reviewed in Chao et al. (2014) in the literature may offer similar information, our new metric (*g*) has an advantage that synthesized information from cohorts such as AGP dataset of 1473 individuals.

### 3.3 Beta‐DAR analysis

Previous alpha‐DAR analysis shows that 100 times of sampling operations are large enough to deal with the random noise from area accrual. I then only sampled 100 times to conduct beta‐DAR analysis to save computational resources (I observed that the computing load of beta‐diversity analysis is nearly 10 times that for alpha‐diversity), and the results are listed in Table 3. The symbols in Table 3 are the same as those in previous Tables 1 and 2 of alpha‐DAR analysis. From Tables 3, I obtain the following findings regarding the test of beta‐DAR models. Overall, the findings from beta‐DAR are rather similar to those from alpha‐DAR, and therefore, I keep the exposition of Table 3 intentionally brief.

#### 3.3.1 The performance of beta‐DAR models

The goodness‐of‐fittings of the three DAR models (PL, PLEC, and PLIEC) to the beta‐diversity scaling with the AGP dataset are even slightly better than to the alpha‐diversity scaling. For example, the minimum percentage of successfully beta‐DAR models is 93% in Table 3, compared with 90% in Tables 1 and 2. The minimum of average *R* (linear correlation coefficients) in beta‐DAR models (Table 3) is 0.555, higher than that of 0.465 in alpha‐DAR models (Table 1). Therefore, beta‐diversity scaling can be modeled with the same mathematical functions as alpha‐diversity scaling models. To the best of our knowledge, this is the first systematic modeling of the scaling of beta‐diversity in the Hill numbers.

Similar to the previous alpha‐DAR model, I also counted the negative *z*‐values when the three DAR models were fitted to beta‐diversity scaling and the results are listed in Table 4 (the same table as for alpha‐DAR). The percentages of negative *z* of the three models PL, PLIEC, and PLEC at *q *=* *2 for beta‐diversity DAR are 24%, 40%, and 14% respectively, and at *q *=* *3, 29%, 42% and 22%, respectively. These percentages are somewhat higher than their alpha‐diversity counterparts discussed previously, but our explanations and conclusions are the same as those previously summarized and recommended for the alpha‐diversity scaling.

#### 3.3.2 The parameter ranges of beta‐DAR models

The beta‐DAR profile, that is, the *z–q* series with the PL for beta‐diversity scaling is beta‐*z* = [0.311, 0.078, 0.027, 0.019] (Figure 1). This series is rather close to that for alpha‐DAR model, which is alpha‐*z* = [0.315, 0.085, 0.037, 0.020]. Overall, the scaling patterns for both alpha‐DAR and beta‐DAR are rather similar. As existing literature has not established a systematic range for the beta‐diversity scaling, there are no existing studies with which I can compare the range of scaling parameters.

#### 3.3.3 Beta‐MAD‐profile prediction

**Figure 3:**
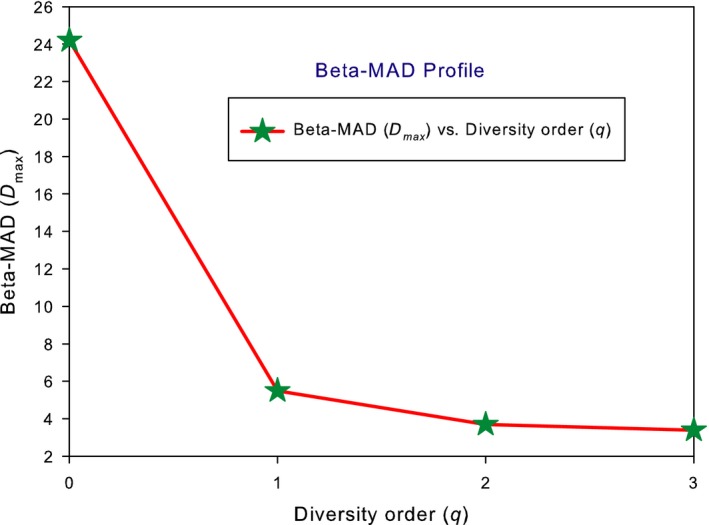
The beta‐MAD profile for the beta‐diversity built with the AGP dataset: The MAD profile is monotonically decreasing with diversity order (*q*)

The beta‐MAD profile predicted by the beta‐DAR PLEC models, that is, *beta‐D*_max_ = [24.3, 5.5, 3.7, 3.4] (Figure 3) and *beta*‐*A*_max_ = [2123, 848, 818, 953] for (*q *=* *0, 1, 2, 3). This beta*‐D*_max_‐*q* series is orders of magnitude smaller than its alpha counterpart, which is *alpha*‐*D*_max_ = [9434, 229.7, 47.4, 24.5], although both the *q*–*A*_max_ series are rather close to each other. The magnitudes of differences in *D*_max_ between alpha‐ and beta‐diversity scaling are, of course, expected because the values of alpha and beta diversities are simply at rather different magnitudes.

#### 3.3.4 Pair‐wise beta‐diversity overlap

Similar to pair‐wise alpha‐diversity overlap, I obtained the *g*‐series (*q *=* *0–3) or PDO profile for beta‐DAR is *beta‐g *= [0.759, 0.944, 0.981, 0.987] (Figure 1), which is rather close to that for the alpha‐DAR, that is, *alpha‐g *= [0.756, 0.939, 0.976, 0.987]. This indicates that while the values of alpha‐diversity and beta‐diversity are at different orders of magnitudes, the degree (level) of their pair‐wise diversity overlaps is essentially independent of the type of diversity measure adopted (alpha or beta).

#### 3.3.5 Summary on the beta‐DAR

When measured in the Hill numbers, the beta‐diversity follows the same scaling law as the alpha‐diversity does. Indeed, both alpha‐DAR and beta‐DAR follow the same scaling law as the traditional SAR does. This finding should be expected if I realize that all Hill numbers (either for measuring alpha, beta, or gamma diversities) are in units of *species* (or as species equivalents), and measure the effective number of species. Indeed, it was this fundamental property of the Hill numbers that motivated us to extend SAR to general DAR. In other words, SAR is a special case of DAR when the diversity order is set to zero (i.e., species richness when *q *=* *0). The tests with the AGP dataset verified our postulation that motivated this study.

## 4 DISCUSSION

Multiple mechanisms have been proposed to explain the classic SAR, including more individuals (also known as passive sampling, random placement, rarefaction effect, sampling effect, etc.), environmental heterogeneity (spatial or temporal), dispersal limitations, population dynamics, niche‐based interactions, biotic interactions, multiple species pools, meta‐population theory, and self‐similarity (*see* reviews by White et al. (2006), Scheiner et al. (2011)). In spite of the extensive studies in macroecology, little direct experimental evidence exists in the literature to prove or reject those proposed mechanisms. Unlike many physical laws whose mechanisms can be theoretically derived and experimentally verified, ecological laws are usually established inductively by the accumulation of experimental data. Although the accumulated ecological data may establish the validity of an ecological law, the data that can *directly* determine or reveal the mechanism are frequently difficult to collect. Due to this limitation, meta‐analysis is often used to investigate the factors (variables) that may affect ecological law. In the case of SAR, quite a few excellent meta‐analysis or similar synthesis (not necessarily followed meta‐analysis procedure strictly) studies exist (e.g., Drakare et al., 2006), but the results of meta‐analysis usually only identify the factors that significantly affect ecological laws (SAR), still may not offer direct evidence to support or reject a specific mechanism hypothesis underlying the law because the complex interaction among the factors is usually hard to consider in meta‐analysis, which may play an important role in controlling the behavior of ecosystem (or community). This somewhat unique property of ecological laws also explains why I cannot offer definite conclusion on the mechanism underlying the DAR of the human microbiome. For example, Drakare et al. (2006) meta‐analysis with 794 SAR studies reported in major ecological journals revealed that SAR is significantly influenced by variables determining sampling schemes, the spatial scale, and the types of organisms or habitats involved. Those meta‐analyses on SAR also offered important insights on the model selection (more than 20 SAR models have been proposed, tested, and evaluated) and other important issues (Tjørve, 2009; Triantis et al., 2012; Williams et al., 2009). Our study benefits enormously, especially on the study design including the model selection and fitting, choice of sampling scale (unit), accrual scheme, from the insights and recommendations reported in those existing meta‐analyses. Even with these efforts, like many other SAR studies, I could not escape from the general limitation involved in the research of ecological laws.

As demonstrated in previous sections, I systematically extend the traditional SAR relationships to their counterparts of DAR relations for both alpha‐diversity and beta‐diversity, based on the known most appropriate diversity metrics—the Hill numbers. These extensions enrich our tools for investigating the biogeography of ecological communities and ecosystems in general, which can be particularly true for deepening our understanding of the biogeographic properties such as spatial heterogeneity of the human microbiome (e.g., Hanson, Fuhrman, Claire Horner‐Devine, & Martiny, 2012; Oh et al., 2014). The DAR models are likely to offer important guidelines for conserving arguably the most important biodiversity to our health—the diversity of our gut microbiome (O'Doherty et al., 2014), similar to the role of SAR in conservation biology.

It should be pointed out that the focus of the present article was centered on the definitions and computational procedures (methodology) for extending the classic SAR to more general DAR. I intentionally chose a large, but with relatively simple sampling design, dataset of the American gut project, to simplify the demonstration of the extensions. In a follow‐up, more application‐oriented study (Ma, 2018), we utilized more comprehensive and sophisticated datasets from the HMP (human microbiome project), which includes samples from 18 body sites of a cohort of 242 individuals. Some of the DAR features, including their biological interpretations, may be better illustrated in the follow‐up application reported in Ma (2018). Nevertheless, I should emphasize that the concepts and estimations of PDO and MAD, especially those of MAD, are rather complex, and cautions must be taken when they are recommended for practical applications. This is because additional factors beyond those considered in building DAR models may influence their estimates, because, ultimately, MAD depends on the parameters of PLEC‐DAR models, and PDO depends on the scaling parameter of PL‐DAR. In particular, MAD‐*D*_max_ depends on all three parameters (*z, c*, and *d*) of PLEC model, while PDO‐*g* only depends on the scaling parameter (*z*) of the PL model. I expect that the parameter *c* is likely influenced by sampling schemes adopted (especially sampling unit or scale). In the case of microbial DAR, sequencing platforms including bioinformatics software pipelines may have an effect on the estimation of parameter *c*. A reason I am less concerned with the estimation of scaling parameter (*z*) is to do with the property of the power law model, which is scale invariant as explained in Ma (2015).

## CONFLICT OF INTEREST

None declared.

## AUTHOR CONTRIBUTION

MZS conceived the idea, designed the study, performed the computation with some external help as stated in the acknowledgement and wrote the manuscript.

## DATA ACCESSIBILITY

The AGP dataset is available at: https://github.com/biocore/American-Gut/tree/master/data/AG
