# Supplementary material for: DAR (diversity–area relationship): Extending classic SAR (species–area relationship) for biodiversity and biogeography analyses
Source: Ecol Evol. 2018 Sep 25;8(20):10023–38. doi: 10.1002/ece3.4425 (PMC6206192; doi:10.1002/ece3.4425)
Supplement: Supplementary file 2 [file ECE3-8-10023-s002.pdf]

Supplementary Documents (I) for "DAR (diversity-area relationships): extending classic SAR (species-area relation) for biodiversity and biogeography analyses"

List of Supplementary Documents
Supporting information to Section of "Material and Method" (Sub-section of Extending SAR to DAR)
Supporting information to Section of "Results" (Supplementary Tables S1-S3)

Supporting information to "Extending SAR to DAR" Sub-section

To save page space, the methods description in the main document was kept brief, and more detailed description on the computational models and procedures for extending SAR to DAR is provided as follows.

(*i*) Definitions of alpha and beta diversities. We use the Hill numbers to measure both alpha and beta diversities, and we adopt *multiplicative version* of the Hill numbers as beta diversities.

The Hill numbers, originally introduced as an *evenness* index from economics by Hill (1973) who was apparently inspired by Renyi's (1961) general entropy of order, has not received the attention it deserves until recent years. Chao et al. (2012) further clarified Hill's numbers for measuring alpha diversity as (with slightly different symbols and notations):

$$^{q}D = \left(\sum_{i=1}^{S} p_i^q\right)^{1/(1-q)} \qquad (1)$$

where *S* is the number of species, *q* is the order number.

The Hill number is undefined for *q*=1, but its limit as *q* approaches to *1* exists in the following form:

$$^{1}D = \lim_{q\to 1} {^{q}D} = \exp\left(-\sum_{i=1}^{s} p_i \log(p_i)\right) \qquad (2)$$

The parameter *q* determines the sensitivity of the Hill number to the relative frequencies of species abundances. When *q*=0, the species abundances do not count at all and $^{0}D=S$, *i.e.,* species richness. When *q*=1, ^{1}D equal the *exponential* of Shannon entropy, and is interpreted as the number of typical or common species in the community. When *q*=2, ^{2}D equal the reciprocal of Simpson index, *i.e.*,

$$^{2}D = (1/\sum_{i=1}^{S} p_i^2) \qquad (3)$$

which is interpreted as the number of dominant or very abundant species in the community (Chao et al. 2012).

The general interpretation of diversity of order *q* is that the community contains $^{q}D=x$ equally abundant species. Then, the diversity of a community can be measured with a series of Hill numbers, possibly plotted on a single graph as a 'continuous' function of the parameter *q*. Chao et al (2012) termed the series of plots "*community diversity profile*" that characterizes the species-abundance distribution of a community and offers complete information on its diversity. Since all Hill numbers are in units of species, and in fact, they are referred to as the effective number of species or as species equivalents, therefore, intuitively, Hill numbers should follow the same or similar pattern of SAR.

Recent studies (*e.g.*, Jost 2007, Ellison 2010, Chao et al. 2012, Gotelli & Chao 2013) have advocated the use of multiplicatively defined beta-diversity, rather than additively defined, by partitioning gamma diversity

into the product of alpha and beta, in which both alpha and gamma diversities are measured with the Hill numbers. For example, Jost (2007) demonstrated that the partition of Hill numbers into independent alpha (within community) and beta (between communities) is necessarily multiplicative such as:

$$^{q}D_{\beta} = {}^{q}D_{\gamma} / {}^{q}D_{\alpha} \quad (4)$$

This beta diversity derived from the above partition takes the value of 1 if all communities are identical, the value of N (the number of communities) when all the communities are completely different from each other (there are no shared species). With Whittaker (1972) words, this beta diversity measures "*the extent of differentiation of communities*;" or with Jost (2007) words, "*the effective number of completely distinct communities*." Indeed, recent advances (Jost 2007, Ellison 2010, Chao 2012, Chao et al. 2014, Chiu et al. 2014, Chao & Jost 2015) have made a convincing case that Hill numbers and multiplicative beta-partition offer to date the most generally consistent and appropriate, yet simple solution to investigate diversity. For this we stick to the Hill numbers and multiplicative beta-diversity in this study.

To compute the Hill numbers of beta diversity with Eq. (4), we first must compute the Hill numbers of gamma diversity of multiple local communities (regional or metacommunity). Let us consider a fixed set of N local communities. Assume that there are S species in the pooled assemblages (meta-community), y_{ij} is the abundance of the i-th species in the j-th local community. Let $y_{++} = \sum_{i=1}^{S}\sum_{j=1}^{N} y_{ij}$ be the total abundance in the meta-community, and let $y_{+j} = \sum_{i=1}^{S} y_{ij}$ be the community size of the j-th local community. Then we may denote the value y_{ij} as $y_{ij} = y_{++}(y_{+j}/y_{++})(y_{ij}/y_{+j}) = y_{++}w_j p_{ij}$, where $p_{ij} = y_{ij}/y_{+j}$ is the relative abundance of the i-th species in the j-th community, and $w_j = y_{+j}/y_{++}$ is the relative community size or the weigh of the j-th community, with $\sum_{j=1}^{N} w_j = 1$. For the gamma community, we pool the species abundances across local communities, and let $y_{i+} = \sum_{j=1}^{N} y_{ij} = y_{++}\sum_{j=1}^{N} w_j p_{ij}$ be the total value of the i-th species in the metacommunity. Then the alpha diversity and gamma diversity of the meta-community can be computed as (5) & (6) respectively,

$$^{q}D_{\alpha} = \frac{1}{N}\left\{\sum_{i=1}^{S}\sum_{j=1}^{N}\left(\frac{y_{ij}}{y_{++}}\right)^{q}\right\}^{1/(1-q)} = \frac{1}{N}\left\{\sum_{i=1}^{S}\sum_{j=1}^{N}\left(w_j p_{ij}\right)^{q}\right\}^{1/(1-q)} \quad (q \neq 1) \quad (5)$$

$$^{q}D_{\gamma} = \left\{\sum_{i=1}^{S}\left(\frac{y_{i+}}{y_{++}}\right)^{q}\right\}^{1/(1-q)} = \left\{\sum_{i=1}^{S}\left(\sum_{j=1}^{N} w_j p_{ij}\right)^{q}\right\}^{1/(1-q)} \quad (q \neq 1). \quad (6)$$

When q=1, the corresponding $^{1}D_{\alpha} = \lim_{q\to 1} {}^{q}D_{\alpha}$ and $^{1}D_{\gamma} = \lim_{q\to 1} {}^{q}D_{\gamma}$, and their detailed formulae can be found in Chiu et al. (2014) and we omit them here.

In this article, we compute diversities until q=3, *i.e.,* to the third order, which includes traditional *species richness* (q=0), the exponential of Shannon index (q=1), the reciprocal of Simpson index (q=2) and one additional set of indexes for q=3.

(*ii*) The DAR models and DAR profiles

See the main text, no supplements needed.

(*iii*) Sampling schemes to fit DAR models

Multiple sampling schemes or types of SAR have been devised to test the SAR theory including *strictly nested* (Type I), *contiguous quadrats grid* (Type-II), *non-contiguous quadrats grid* (Type-III), and *areas of*

varying size (*islands*, Type-IV) (Scheiner et al. 2003, 2011). The first three types are termed *aggregate* SARs and the fourth type is termed independent SAR. According to Whittaker & Triantis (2012), the most critical distinction is whether cumulative totals are computed *across a set of areas* or whether the actual number of species detected in each area is *directly* utilized for constructing SAR. Accordingly, some researchers refer to the former as the *species accumulation curves* (SACs) and the latter as *island species area-relationships* (ISARs). We use the more general and popular term species-area relationship (SAR) in the study, although our approach belongs to the former category (*i.e.*, SAC). It is noted that *island* (Type-IV) appears to be a reasonable sampling scheme in our study, but the islands in the human microbiome are obviously of *approximately* the same size. According to the above-mentioned Whittaker & Triantis (2012) distinction, the *island* model is hardly applicable to the study of human microbiome.

In this study, we adopt the Scheiner (2003, 2011) Type-III sampling scheme, non-contiguous quadrats grid. Strictly speaking, quadrats (the same size) are arranged in a regular but noncontiguous grid. Since the quadrat (individual subject), *i.e.,* our body (habitats for our microbiome) is highly mobile, figuring out a definite spatial coordinate relationship, not to mention a grid of quadrats, is hardly possible. Nevertheless, Type-III is obviously the most natural choice we can make. To deal with the lack of spatial order (grid), we adopt the following three-step scheme. *Step* (*a*): generate all possible sequences of the subjects, *i.e.,* the total permutations of all subjects in the HMP dataset. *Step* (*b*): randomly choose 100 (or 1000) sequences (as 100 or 1000 repetitions) from the total permutations, and each (sequence) of the 100 (1000) sequences of the subjects is treated as a 'grid' of subjects. Each 'grid' of subjects is utilized to accumulate diversity and fit a DAR curve. *Step* (*c*): the average parameters from the 100 (1000) repetitions (DAR curves) are adopted as the model parameters of the final DAR model selected for the dataset.

Here, we further explain our sampling scheme for constructing DAR models. As explained previously, unlike most studies in macro-ecology, where there is often a natural spatial sequence (or arrangement) among the communities sampled, the community samples in our HMP data are pretty much 'random.' In other words, there is not a naturally occurring spatial sequence (arrangement) among the communities of the HMP samples we use for modeling DAR. To avoid the potential bias from an arbitrary order of the community samples, we totally permutated the orders of all the community samples under investigation, and then randomly choose 100 (or 1000) orders of the communities generated from the permutation operation. That is, rather than taking an arbitrary order for accruing community samples in one-time fitting to the DAR model, we repeatedly perform the DAR model-fitting 100 (1000) times, each time the community samples used to build the DAR model was randomly chosen from the total permutation of all the community samples under investigation. It is noted that the 100 (1000) times of sampling from the total permutation were conducted randomly without repetition (replacement), *i.e*., a specific permutation is used at most once for DAR model fitting. Finally, the averages of the model parameters from the 100 (1000) times of DAR fittings are adopted as the model parameters of the DAR for the set of community samples under investigation.

Occasionally, there may be the cases when the DAR model parameters do not make sense ecologically. For example, in the case of PLEC model [eqn. (6)], the sign of parameter z and d must be opposite to have $A_{\max}>0$ [eqn. (11)], otherwise, $A_{\max}<0$ the model does not have an extreme value. In this kind of exceptional case, we simply discard the specific permutation that generated the exception, and add another round of model fitting by randomly taking another order of samples from the total permutation; the additional fittings may be repeated as needed until a positive exponent is obtained. Note that we only remove exceptions that do not make sense ecologically such as $A_{\max}<0$ (negative number of accrual is not possible). For another example, again in the case of PLEC, negative z and positive d are possible for DAR beyond species richness (q=0), which are considered as valid permutations.

(*iv*) The accrual of diversities to fit DAR models

After selecting the sampling schemes, we need to specify the ***scheme to accrue diversity*** based on the diversity formulae listed above [Eqns. (1-6)]. Although the accumulation of species in traditional SAR is well defined and there is no ambiguity on how the species counts are computed once the scheme for area accrual is decided, the computation of the diversity accrual is still largely an uncharted area, and there may be more than one scheme to accumulate diversity across space, especially for the accrual of beta-diversity. To devise what we believe to be the most appropriate and also natural methods to accrue diversity, we follow the following three principles. The first is to use the Hill numbers, or what Jost (2007) termed the *true* diversity; the second is to follow the essence of SAR, as captured by the word "accumulation" or "aggregate," *i.e.*, species (diversity) are accumulated for the accrued areas; the third is that the diversity scaling model should be useful for *predicting* diversity at different levels of areas accumulated. We consider these three principles as axioms in traditional SAR and we believe that any extension from SAR to DAR should not violate them.

The last principle is a major reason why SAR has been a central theme of both community ecology and conservation biology, and is obviously important for understanding the biogeography of human microbiome. One important advantage for us to stick to the three principles, which are embodied in the traditional SAR theory, is that our new DAR should inherit many of the insights and applications that traditional SAR has reveled and offered. Based on these three principles, we construct the following procedures to accumulate diversity over areas.

To accrue alpha diversity across areas, at each accrual step, we *first* simply add up all the OTU lists (rows) in the OTU table of the communities up to the accrual step. Assuming there are N communities, at accrual step i=1, 2,…, N-1, we simply add up the rows in the standard OTU table corresponding to the first i communities, until the last row is added when the accrual is completed. For each of the aggregate (accumulated) community, we compute its alpha-diversity with Eq. (1) or Eq. (2) (for q=1) with the added-up OTU lists for the accrual step. The resulted pairs of accrued alpha diversities and areas are regressed to fit the DAR model.

An alternative accrual scheme is to use Eq. (5), but the output with Eq. (5) would be a measure of average single community (local community) diversity, which is not a cumulative quantity and is hence not a measure that we are interested in to investigate DAR relationship. This is because Eqn. (5) is constrained by its mission to partition gamma diversity [Eq. (6)] multiplicatively into alpha diversity [Eq. (5)] and beta-diversity [Eq. (4)]. Because it is a measure of the *average* alpha diversity of the local communities that constitute the regional meta-community, rather than the alpha-diversity of aggregate (pooled or accumulated) communities, therefore, this alternative scheme contradicts the second and third principles we identified above.

Yet another alternative accrual scheme is to use Eq. (6), which calculates the gamma diversity of pooled (aggregate or accumulated) regional or meta-community. Although conceptually it computes the gamma diversity of the aggregate community, it is actually equal to the alpha diversity we calculated for the aggregate community with Eqns. (1) or (2) (when q=1) when the community weight (w_j) is equal for all N communities (*i.e.*, w_j=1/N). Since the equal weight community assumption is largely true even if the weights are not exactly equal, we can expect that the difference between the results from Eqns. (1) and (6) should not to have significant influence on DAR modeling results. Therefore, we do not see an advantage with this alternative accrual scheme, especially the potential confusion in terminology. In this case, whether it is called alpha or gamma diversity matters little, and from the perspective of SAR (DAR), we prefer to use the term alpha-diversity.

To the best of our knowledge, scaling of beta-diversity has not been approached in the existing literature. Therefore, we have a benefit to prioritize the potential utilization that the beta-diversity scaling relationship may possess. We believe that the three axioms (principles) we identified above should also guide the extension of SAR to beta-DAR. Furthermore, the capability to predict beta-diversity of aggregate community, as designated in principle 3, is also the priority that the beta-DAR should pursue, in our belief. Through trial-and-error exploration, we found that the following scheme for accruing beta-diversity over areas best satisfy the three principles we set for developing beta-DAR.

Formally, to accrue beta-diversity across areas, we start the computational procedure with two local communities (samples) by using the formulae specified by Eq. (4)-(6), from which the first beta-diversity value is computed for the two initial communities. For each newly added community (sample) at the accrual step *i* (*i=3, 4, ... N*), we simply run the same computation procedure (same equations) with *3*, *4*, … *N* samples, until all *N* communities are accrued for their beta-diversity. With each newly added local community, we obtain a new Hill numbers of beta-diversity, until all *N* communities are accrued for their beta-diversity. The series of beta-diversities are regressed with their respective areas accrued at each step. Obviously, this accrual scheme calculates the *accumulated beta-diversity* of *N* communities (of individuals in the case of human microbiome). It is also the *maximal* difference among *N* communities in terms of beta-diversity.

(*v*) Predicting MAD (Maximal Accrual Diversity) with PLEC-DAR models
No supplement to this sub-section is need.

(***vi***) **Self-similarity property of PL-DAR and the prediction of pair-wise diversity overlap**
No supplement to this sub-section is need.

Supporting information to Section of "Results" (Tables S1-S3):

Table S1. Fitting the alpha-Diversity Area Relationship (alpha-DAR) with 100 times of re-sampling of the 1473-Subjects AGP datasets (without removing any failed fitting cases to calculate the statistics of model parameters)

Diversity Order (q) and Statistics		Power Law (PL)					PL with Inverse Exponential Cutoff (PLIEC)					PL with Exponential Cutoff (PLEC)						
		z	ln(*c*)	*R*	*p*-value	*g*	*z*	*d*	ln(*c*)	*R*	*p*-value	*z*	*d*	ln(*c*)	*R*	*p*-value	A_{max}	D_{max}
q=0	Mean	0.315	6.908	0.986	0.000	0.756	0.291	-1.493	7.067	0.996	0.000	0.387	-0.0002	6.593	0.995	0.000	1994	9407.4
	Std. Err.	0.001	0.005	0.000	0.000	0.001	0.001	0.024	0.006	0.000	0.000	0.001	0.0000	0.007	0.000	0.000		
	Min	0.297	6.775	0.975	0.000	0.742	0.271	-2.211	6.950	0.990	0.000	0.347	-0.0003	6.387	0.988	0.000		
	Max	0.332	7.023	0.993	0.000	0.771	0.308	-0.946	7.214	0.999	0.000	0.429	-0.0001	6.809	0.999	0.000		
q=1	Mean	0.085	4.849	0.789	0.000	0.939	0.058	-1.677	5.027	0.930	0.000	0.165	-0.0002	4.504	0.900	0.000	775	229.2
	Std. Err.	0.002	0.014	0.009	0.000	0.001	0.002	0.054	0.016	0.004	0.000	0.003	0.0000	0.018	0.006	0.000		
	Min	0.044	4.475	0.529	0.000	0.900	0.010	-3.350	4.624	0.816	0.000	0.081	-0.0004	4.100	0.654	0.000		
	Max	0.138	5.130	0.943	0.000	0.969	0.115	-0.599	5.357	0.987	0.000	0.239	-0.0001	4.935	0.988	0.000		
q=2	Mean	0.034	3.611	0.459	0.051	0.976	0.014	-1.207	3.740	0.763	0.000	0.085	-0.0001	3.390	0.658	0.007	622	47.0
	Std. Err.	0.003	0.020	0.028	0.018	0.002	0.003	0.061	0.023	0.014	0.000	0.005	0.0000	0.026	0.023	0.007		
	Min	-0.012	3.040	0.001	0.000	0.917	-0.047	-2.764	3.125	0.431	0.000	-0.017	-0.0003	2.825	0.024	0.000		
	Max	0.115	3.959	0.955	0.978	1.008	0.102	0.035	4.191	0.971	0.000	0.193	0.0001	3.919	0.976	0.661		
q=3	Mean	0.019	3.053	0.438	0.028	0.987	0.005	-0.846	3.143	0.667	0.000	0.052	-0.0001	2.907	0.601	0.000	586	24.3
	Std. Err.	0.003	0.021	0.025	0.013	0.002	0.003	0.060	0.023	0.020	0.000	0.005	0.0000	0.027	0.022	0.000		
	Min	-0.030	2.512	0.001	0.000	0.931	-0.056	-2.235	2.512	0.109	0.000	-0.064	-0.0003	2.308	0.083	0.000		
	Max	0.096	3.423	0.956	0.969	1.021	0.096	0.566	3.584	0.961	0.000	0.169	0.0002	3.517	0.963	0.006		

Table S2. Fitting the alpha-Diversity Area Relationship (alpha-DAR) with 1000 times of re-sampling of the 1473-Subjects AGP datasets (without removing any failed fitting cases to calculate the statistics of model parameters)

q		Power Law (PL)					PL with Inverse Exponential Cutoff (PLIEC)					PL with Exponential Cutoff (PLEC)						
		z	ln(*c*)	*R*	*p*-value	*g*	*z*	*d*	ln(*c*)	*R*	*p*-value	*z*	*d*	ln(*c*)	*R*	*p*-value	A_{max}	D_{max}
q=0	Mean	0.314	6.911	0.986	0.000	0.757	0.290	-1.508	7.071	0.996	0.000	0.386	-0.0002	6.598	0.995	0.000	2006	9413.6
	Std. Err.	0.000	0.002	0.000	0.000	0.000	0.000	0.008	0.002	0.000	0.000	0.000	0.0000	0.002	0.000	0.000		
	Min	0.291	6.771	0.954	0.000	0.739	0.262	-3.261	6.917	0.989	0.000	0.336	-0.0003	6.295	0.978	0.000		
	Max	0.335	7.081	0.995	0.000	0.777	0.312	-0.847	7.269	0.999	0.000	0.456	-0.0001	6.866	0.999	0.000		
	p-Norm.	0.234	0.207				0.002	0.000	0.000			0.115	0.004	0.561				
q=1	Mean	0.082	4.870	0.784	0.000	0.942	0.054	-1.714	5.051	0.935	0.000	0.157	-0.0002	4.542	0.890	0.000	780	228.6
	Std. Err.	0.001	0.004	0.003	0.000	0.000	0.001	0.020	0.005	0.001	0.000	0.001	0.0000	0.007	0.002	0.000		
	Min	0.016	4.438	0.350	0.000	0.895	-0.007	-5.275	4.568	0.738	0.000	0.022	-0.0004	3.861	0.412	0.000		
	Max	0.144	5.286	0.958	0.000	0.989	0.125	-0.339	5.469	0.993	0.000	0.290	0.0000	5.263	0.993	0.000		
	p-Norm.	0.772	0.698				0.397	0.000	0.465			0.138	0.044	0.832				
q=2	Mean	0.028	3.645	0.446	0.023	0.980	0.009	-1.224	3.775	0.749	0.000	0.072	-0.0001	3.456	0.635	0.002	617	46.8
	Std. Err.	0.001	0.006	0.008	0.004	0.001	0.001	0.023	0.007	0.005	0.000	0.002	0.0000	0.010	0.007	0.001		
	Min	-0.068	2.942	0.001	0.000	0.905	-0.088	-4.339	3.025	0.083	0.000	-0.102	-0.0004	2.570	0.023	0.000		
	Max	0.131	4.301	0.956	0.973	1.046	0.118	0.499	4.438	0.983	0.006	0.245	0.0002	4.403	0.966	0.673		
	p-Norm.	0.000	0.000				0.001	0.000	0.001			0.000	0.001	0.060				
q=3	Mean	0.013	3.090	0.401	0.030	0.991	-0.001	-0.859	3.181	0.653	0.001	0.039	-0.0001	2.978	0.575	0.001	561	24.2
	Std. Err.	0.001	0.007	0.008	0.004	0.001	0.001	0.023	0.007	0.007	0.000	0.002	0.0000	0.010	0.007	0.001		
	Min	-0.098	2.345	0.000	0.000	0.914	-0.108	-3.746	2.403	0.047	0.000	-0.153	-0.0004	2.028	0.024	0.000		
	Max	0.119	3.842	0.957	0.996	1.065	0.112	0.901	3.906	0.976	0.203	0.213	0.0003	3.985	0.971	0.656		
	p-Norm.	0.000	0.000				0.001	0.000	0.002			0.000	0.000	0.028				

Table S3. Fitting the beta-Diversity Area Relationship (beta-DAR) with 100 times of sampling of the 1473-Subjects AGP datasets (without removing any failed fitting cases to calculate the statistics of model parameters)

Diversity Order (q) and Statistics		**Power Law (PL)**					**PL with Inverse Exponential Cutoff (PLIEC)**					**PL with Exponential Cutoff (PLEC)**						
		z	**ln(*c*)**	***R***	***p*-value**	***g***	***z***	***d***	**ln(*c*)**	***R***	***p*-value**	***z***	***d***	**ln(*c*)**	***R***	***p*-value**	A_{max}	D_{max}
q=0	Mean	0.311	0.971	0.990	0.000	0.759	0.283	-2.236	1.155	0.997	0.000	0.377	-0.0002	0.683	0.996	0.000	2056	24.2
	Std. Err.	0.001	0.006	0.000	0.000	0.001	0.001	0.024	0.008	0.000	0.000	0.001	0.0000	0.008	0.000	0.000		
	Min	0.285	0.781	0.981	0.000	0.732	0.251	-2.848	0.963	0.992	0.000	0.338	-0.0003	0.475	0.993	0.000		
	Max	0.343	1.148	0.994	0.000	0.781	0.314	-1.789	1.376	1.000	0.000	0.417	-0.0001	0.885	0.999	0.000		
q=1	Mean	0.078	1.167	0.808	0.000	0.944	0.048	-2.429	1.367	0.951	0.000	0.145	-0.0002	0.878	0.910	0.000	782	5.5
	Std. Err.	0.002	0.015	0.011	0.000	0.002	0.003	0.048	0.017	0.003	0.000	0.003	0.0000	0.019	0.007	0.000		
	Min	0.022	0.833	0.439	0.000	0.908	-0.011	-3.742	0.963	0.871	0.000	0.055	-0.0004	0.502	0.595	0.000		
	Max	0.126	1.554	0.957	0.000	0.984	0.107	-1.412	1.806	0.987	0.000	0.213	0.0000	1.410	0.993	0.000		
q=2	Mean	0.027	1.118	0.560	0.008	0.981	0.004	-1.787	1.265	0.770	0.000	0.073	-0.0001	0.912	0.696	0.004	610	3.7
	Std. Err.	0.004	0.029	0.025	0.006	0.003	0.005	0.094	0.033	0.015	0.000	0.006	0.0000	0.035	0.022	0.004		
	Min	-0.073	0.545	0.015	0.000	0.919	-0.119	-4.999	0.604	0.122	0.000	-0.099	-0.0004	0.177	0.036	0.000		
	Max	0.112	1.826	0.937	0.567	1.050	0.102	0.438	2.124	0.945	0.000	0.195	0.0002	1.823	0.967	0.393		
q=3	Mean	0.018	1.082	0.550	0.002	0.987	-0.001	-1.534	1.209	0.701	0.000	0.061	-0.0001	0.883	0.688	0.000	605	3.4
	Std. Err.	0.005	0.036	0.025	0.002	0.004	0.006	0.125	0.043	0.017	0.000	0.009	0.0000	0.047	0.021	0.000		
	Min	-0.123	0.404	0.037	0.000	0.914	-0.178	-6.124	0.478	0.205	0.000	-0.192	-0.0005	0.040	0.129	0.000		
	Max	0.119	1.984	0.923	0.157	1.082	0.107	1.008	2.389	0.933	0.000	0.209	0.0004	2.272	0.958	0.000		

References for the Supplementary Document

Chao A, CH Chiu, TC Hsieh (2012) Proposing a resolution to debates on diversity partitioning. *Ecology*, 93(9): 2037-2051.

Chao A, CH Chiu, & L Jost (2014) Unifying species diversity, phylogenetic diversity, functional diversity and related similarity and differentiation measures through Hill numbers. *Annual Reviews of Ecology, Evolution, and Systematics*, 45:297–324.

Chao A, NG Gotelli, TC Hsieh, EL Sander, KH Ma, RK Colwell, & AM Ellison (2014) Rarefaction and extrapolation with Hill numbers: a framework for sampling and estimation in species biodiversity studies. *Ecological Monographs*, 84, 45–67.

Chao, A and L Jost (2015) Estimating diversity and entropy profiles via discovery rates of new species. *Methods in Ecology and Evolution* 2015, doi: 10.1111/2041-210X.12349

Chiu CH, L Jost, A Chao (2014) Phylogenetic beta diversity, similarity, and differentiation measures based on Hill numbers. *Ecological Monographs*, 84(1): 21-44.

Ellison AM (2010) Partitioning diversity. *Ecology* 91:1962– 1963.

Gotelli, N.J. & Chao, A. (2013) Measuring and estimating species richness, species diversity, and biotic similarity from sampling data. Encyclopedia of Biodiversity, vol 5, 2nd ed. (ed. S.A. Levin), pp. 195–211. Academic Press, Massachusetts, USA.

Gotelli, NJ & AM Ellison (2013) *A Primer of Ecological Statistics*. Sinauer Associates Inc. 2ed.

Hill MO (1973) Diversity and evenness: a unifying notation and its consequences. *Ecology,* 54:427-342.

Jost L (2007) Partitioning diversity into independent alpha and beta components. *Ecology* 88:2427–2439.

Renyi A (1961) On measures of entropy and information, p. 547-561. Neyman, ed. 4th *Berkeley symposium on mathematical statistics and probability*. Berkeley.

Scheiner SM (2003) Six types of species–area curves. *Global Ecology and Biogeography* 12:441–447.

Scheiner SM, A Chiarucci, GA Fox, MR Helmus, DJ McGlinn, MR Willig (2011) The underpinnings of the relationship of species richness with space and time. *Ecological Monographs* 81(2):195-213.

Whittaker, RH (1972) Evolution and measurement of species diversity. *Taxon*, 21, 213–251.

Whittaker RJ, and KA Triantis (2012) The species–area relationship: an exploration of that 'most general, yet protean pattern. *J of Biogeography*, 39:623-626
